# Maternal B12, Folate and Homocysteine Concentrations and Offspring Cortisol and Cardiovascular Responses to Stress

**DOI:** 10.1210/clinem/dgz114

**Published:** 2020-03-24

**Authors:** Ghattu V Krishnaveni, Sargoor R Veena, Matt Johnson, Kalyanaraman Kumaran, Alexander Jones, Dattatray S Bhat, Chittaranjan S Yajnik, Caroline H D Fall

**Affiliations:** 1 Epidemiology Research Unit, CSI Holdsworth Memorial Hospital, Mysore, India; 2 MRC Lifecourse Epidemiology Unit, Southampton General Hospital, Southampton, UK; 3 Department of Paediatrics, University of Oxford, Oxford, UK; 4 Diabetes Unit, King Edward Memorial Hospital and Research Center, Pune, India

**Keywords:** B12 deficiency, folate, homocysteine, stress response, cortisol, adolescent

## Abstract

**Context:**

Imbalances in maternal 1-carbon nutrients (vitamin B12, folate) have been shown to be associated with higher offspring cardiometabolic risk markers in India.

**Objective:**

We examined the hypothesis that low plasma vitamin B12 (B12) and high folate and homocysteine concentrations in the mother are associated with higher hypothalamic–pituitary–adrenal axis (cortisol) and cardiovascular responses during the Trier Social Stress Test for Children (TSST-C) in an Indian birth cohort.

**Methods:**

Adolescents (*n* = 264; mean age: 13.6 years), whose mothers’ plasma B12, folate and total homocysteine concentrations had been measured during pregnancy, completed 5-minutes each of public speaking and mental arithmetic tasks in front of 2 unfamiliar “judges” (TSST-C). Baseline and poststress salivary cortisol concentrations were measured. Heart rate, blood pressure, stroke volume, cardiac output, and total peripheral resistance were measured continuously at baseline, during the TSST-C, and for 10 minutes after the TSST-C using a finger cuff; beat-to-beat values were averaged for these periods, respectively.

**Results:**

Maternal low B12 status (plasma B12 < 150 pmol/L) was associated with greater cortisol responses to stress in the offspring (*P* < .001). Higher homocysteine concentrations were associated with greater offspring heart rate response (*P* < .001). After adjustment for multiple comparisons, there were nonsignificant associations between higher maternal folate concentrations and offspring total peripheral resistance response (*P* = .01).

**Conclusion:**

Our findings suggest that maternal 1-carbon nutritional status may have long-term programming implications for offspring neuroendocrine stress responses.

Psychological stress is a risk factor for cardiometabolic, psychiatric and other noncommunicable diseases (NCD) in adults (1). Altered responses of the hypothalamic–pituitary–adrenal (HPA) axis and autonomic cardiovascular systems to stress are thought to contribute to the physiological changes that lead to these conditions. Indeed, studies have shown increased cardiovascular disease risk in individuals with higher stress responses (2).

The developmental origins hypothesis proposes that impaired nutrition during fetal development leads to phenotypic changes that increase vulnerability to cardiovascular disease in later life (3). Maternal nutrients, specifically, nutrients associated with 1-carbon (1-C) metabolism play an important role in fetal neurodevelopment across the life course (4). Cohort studies in India have shown that low vitamin B12 (B12) status in the face of a high folate status in pregnancy may increase maternal (5) as well as offspring NCD risk (6). Findings from the Pune Maternal Nutritional Study led to the nutrient-mediated teratogenesis hypothesis, which proposed that the imbalance of B12 and folate in the mother may be associated with a spectrum of fetal outcomes, including cardiometabolic risk in India (7). We previously tested for evidence of causality within a Mendelian randomization framework and showed that maternal 1-C metabolism (homocysteine) has a causal association with fetal growth (8).

Recent studies using birth weight as a marker of fetal nutrition showed higher HPA axis and cardiovascular sympathetic nervous system responses in relation to lower birth weight (9–12). However, associations of maternal nutritional status with offspring stress responses in humans have not been reported before. The Mysore Parthenon Study was established with the primary aim to examine the long-term effects of maternal nutritional status on cardiometabolic risk factors in the offspring (13). The study has already demonstrated associations between maternal 1-C nutrients and offspring cardiometabolic outcomes, showing that higher maternal folate and homocysteine concentrations were associated with higher childhood insulin resistance and glycaemia in the offspring (14). In the present study, we test the secondary hypothesis that low maternal plasma B12 and high folate and homocysteine concentrations predict increased HPA axis and autonomic cardiovascular stress responses in the offspring.

## Methods

### The Parthenon Study

During 1997–1998, 830 women booking consecutively into the antenatal clinics of the Holdsworth Memorial Hospital (HMH) in Mysore, India, and matching our eligibility criteria (no known history of diabetes, intention to deliver at HMH, singleton pregnancy) underwent detailed anthropometry and blood sampling at 28 to 32 weeks of pregnancy (13). Plasma samples were stored. Maternal supplements during pregnancy were recorded at recruitment, but not subsequently. Six-hundred and sixty-three women who chose to deliver at HMH gave birth to live babies without major congenital anomalies, and detailed neonatal anthropometry was carried out.

### Offspring follow-up

The children were followed up 6 to 12 monthly for detailed anthropometry and cardiometabolic investigations (13). Twenty-five died in childhood and 8 developed major medical conditions. At 13.5 years, 545 adolescent children were available for follow-up. We administered a standard laboratory based stress-test, the Trier Social Stress Test for Children (TSST-C) (15) in 273 of these adolescents representing 4 birth weight categories, including all available offspring of mothers with gestational diabetes mellitus (*n* = 28), selected from those living within Mysore city (*N* = 354).

### TSST-C

Details of the tests have been reported previously (15). The tests were conducted between 2.00 pm and 3.30 pm, for 1 child at a time. A baseline (pretest) salivary sample was collected 10 minutes before the test, after the children had watched a calming video for 5 minutes in a standing position. They then performed 5 minutes each of public speaking (imaginative storytelling) and mental arithmetic tasks (serial subtraction) standing in front of 2 unfamiliar adult “judges.” Further salivary samples were collected at 10, 20, 30, 40, and 70 minutes after stress induction (start of TSST-C) to measure the cortisol response. Systolic and diastolic blood pressure (BP), cardiac output, stroke volume, heart rate and total peripheral resistance (TPR) were measured continuously using a noninvasive, portable hemodynamic monitoring system with appropriately sized finger cuffs (Nexfin, BMeye, Amsterdam, Netherlands). The beat-to-beat values were averaged over 5 minutes for the baseline (pretest video-viewing), public speaking, mental arithmetic, and immediate post-stressor periods. Change in poststress cortisol and cardiovascular parameters from baseline constituted the stress response.

Weight (Salter, Tonbridge, Kent, UK) and height (Microtoise, CMS instruments) were measured; body mass index (BMI) was calculated using the formula weight/height^2^. Information was collected on recent stressful or traumatic situations that might affect stress reactivity. However, none of the children reported any major traumatic events in this period. Pubertal status was assessed using Tanner’s method (16) and was classified as the stage of breast development (in girls) or genital development (in boys). The socioeconomic status of the family was determined using the standard of living index designed by the National Family Health Survey-2 (17). This is a standardized questionnaire-based index, developed for national surveys in India, and is based on information about housing, amenities, and possessions. Higher score indicates higher social class.

The study was approved by the HMH ethics committee; informed written consent from parents and assent from children were obtained.

### Laboratory assays

Assays were carried out at the Diabetes Unit, KEM Hospital, Pune, India. Maternal B12, folate, and total homocysteine (tHcy) concentrations were analyzed using stored plasma samples. Microbiological assays were used for B12 and folate and fluorescence polarization immunoassay (Abbott) for tHcy (18–20). Intra- and inter-assay coefficients of variation were <8% for these assays. Maternal low B12 status was defined as a concentration <150 pmol/L and low folate status as a concentration <7 nmol/L. Hyperhomocysteinema was defined as a tHcy concentration >10 μmol/L. Salivary cortisol concentrations in children were measured using an enzyme-linked immunosorbent assay method (Alpco Diagnostics, Salem, NH, US). The assay sensitivity was 1 ng/mL; inter- and intra-assay coefficients of variation were 10.0% and 6.6%, respectively.

### Statistical methods

Children were assigned to groups on the basis of their mother’s nutrient deficiency status. After log-transformation of cortisol concentrations to satisfy the assumption of normality, between-group differences in cortisol and cardiovascular measures at baseline were analyzed using independent *t*-tests. Mean (standard deviation [SD]) were presented for normally distributed variables and median (interquartile range) for skewed variables in the tables describing these analyses. Multiple linear regression models were used to adjust these associations for age, sex, socioeconomic status, pubertal stage, current BMI, and maternal gestational diabetes mellitus status and maternal BMI during pregnancy. We performed linear mixed-model analysis to examine associations of maternal B12, folate, and tHcy with repeated measures of salivary cortisol and cardiovascular parameters to account for within-group correlations. Salivary cortisol concentrations at all time points and averaged cardiovascular parameters at different stages of the TSST-C, respectively, were included in the models to examine the change in these parameters from baseline after stress induction (stress response). All models were adjusted for the previously listed variables, and cases with incomplete data were excluded. Exposure and outcome variables were converted into SD scores (SDS) before analysis to allow comparison of effects in units of SD change in the stress response per unit of SD change in the maternal exposure. We corrected for multiple comparisons using the Bonferroni correction, which, for 52 hypothesis tests at the 5% level, yields a threshold for statistical significance of *P* = .001. All analyses were performed using STATA v 15.1.

## Results

Maternal and offspring general characteristics are given in Table 1. Maternal B12, folate, and tHcy concentrations were available for 264 of the 269 children who completed the TSST-C. Low B12 status was present in 46% of the mothers, while only about 3% had low folate levels or hyperhomocysteinemia. There was no difference between the cohort children who took part in the TSST-C and those who were not part of the study in maternal 1-C nutrient status, socioeconomic status, or offspring BMI. Maternal BMI was significantly higher among current participants compared to those who were not included in this study (24.2 vs 23.0 kg/m^2^, *P* < .001).

**Table 1.** Characteristics of mothers at 28 to 32 weeks’ gestation and offspring at 13.5 years

Characteristic	*n*	Measure
**Maternal**		
Age (years)^a^	264	24.0 (21.0, 27.0)
BMI (kg/m^2^)	264	24.2 (3.7)
Vitamin B12 (pmol/L)^a^	264	158.0 (120.5, 214.5)
Low vitamin B12 level (<150 pmol/L)^a^	120 (45.5%)	118.0 (103.0, 131.0)
Normal vitamin B12 level^a^	144 (54.5%)	208.0 (177.5, 250.5)
Folate (nmol/L)^a^	264	34.0 (16.2, 50.8)
Low folate level (<7 nmol/L)^a^	8 (3.0%)	5.7 (5.4, 5.9)
Normal folate level^a^	256 (97.0)	35.6 (17.5, 51.0)
Homocysteine (μmol/L)^a^	264	6.0 (5.0, 7.0)
Hyperhomocysteinemia (>10 μmol/L)^a^	9 (3.4%)	10.8 (10.4, 13.3)
Normal homocysteine level^a^	255 (96.6%)	5.8 (5.0, 6.9)
**Offspring**		
Age (years)^a^	264	13.6 (0.1)
Male sex	132 (50.0%)	-
Height (cm)	264	154.2 (7.1)
BMI (kg/m^2^)^a^	264	17.1 (15.7, 19.3)
Socioeconomic status (SLI score)	264	38.1 (6.6)
Baseline salivary cortisol (ng/ml)^a^	263	6.7 (4.9, 9.0)
Baseline systolic blood pressure (mmHg)	244	100.7 (11.7)
Baseline diastolic blood pressure (mmHg)	244	69.4 (7.8)
Baseline heart rate (bpm)	244	106.4 (12.2)
Baseline cardiac output (L/min)	244	4.6 (0.8)
Baseline stroke volume (ml)	244	43.5 (7.9)
Baseline total peripheral resistance (dyn.s/cm^2^)	244	1489.2 (226.8)

Values presented are mean (standard deviation) unless otherwise noted.

Abbreviations: BMI, body mass index; SLI, standard of living.

^a^median (interquartile range).

Both pretest and posttest salivary cortisol measurements were available for 263 children and complete cardiovascular profiles were available for 244 children. Of these, 247 with complete data for all covariates were included for cortisol response models and 229 for cardiovascular response models.

### Associations with cortisol responses to stress

Offspring of mothers with low B12 status had significantly lower cortisol concentrations at baseline compared to those of mothers with normal B12 levels (median: 6.3 ng/mL [interquartile range 4.8–8.2] vs 7.1 ng/mL [5.1–9.8] in the normal B12 group; adjusted *P* = .03) (Table 2). There were no significant associations of maternal folate or homocysteine concentrations with offspring baseline cortisol concentrations.

**Table 2.** Longitudinal offspring cortisol responses to the trier social stress test by maternal B12, folate, and homocysteine category

	Time after Trier Social Stress Test					
Maternal category	Baseline	10 min	20 min	30 min	40 min	70 min
**Vitamin B12 (pmol/L)**						
Low vitamin B12 (<150 pmol/L)	6.3 (4.8,8.2)	9.0 (5.9,13.3)	12.4 (8.3,18.4)	14.1 (8.4,21.5)	11.9 (8.0,18.3)	8.7 (6.0,12.0)
Normal vitamin B12	7.1 (5.1,9.8)	8.8 (5.9,14.6)	11.9 (7.9,19.5)	12.5 (8.4,19.8)	11.6 (8.2,19.2)	8.7 (6.3,13.7)
**Folate (nmol/L)**						
Low folate (<7 nmol/L)	8.4 (6.4,13.5)	11.3 (6.2,22.8)	14.9 (7.7,27.7)	13.4 (8.5,26.1)	12.8 (6.4,25.9)	8.0 (6.5,19.8)
Normal folate	6.6 (4.8,8.9)	9.0 (5.9,14.0)	12.0 (8.0,18.6)	12.9 (8.4,20.6)	11.9 (8.2,18.6)	8.7 (6.2,12.3)
**Homocysteine (μmol/L)**						
Hyperhomocysteinemia (>10 μmol/L)	7.7 (5.7,8.6)	10.9 (9.6,14.6)	16.2 (14.7,23.1)	20.6 (14.3,30.6)	20.3 (14.5,22.9)	11.4 (9.4,15.6)
Normal homocysteine	6.6 (4.8,9.0)	8.8 (5.9,14.1)	11.7 (7.9,18.6)	12.7 (8.3,20.4)	11.6 (7.9,18.3)	8.6 (6.1,12.3)

*N* = 264. All reported values are median (interquartile range).

**Figure 1.**
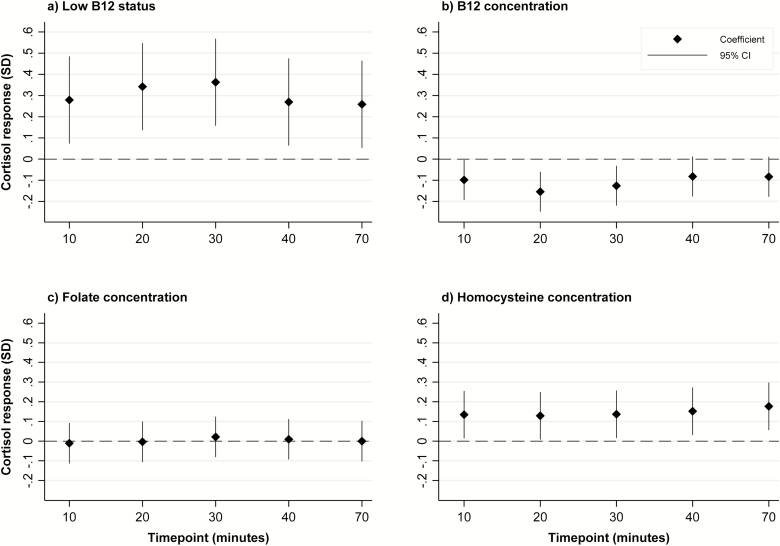
Associations of maternal low B12 status, and maternal B12, folate and homocysteine concentrations with offspring cortisol response to the Trier Social Stress Test. Graph (a): Values represent standard deviation (SD) change from baseline in log-transformed cortisol response for the maternal low B12 status group, compared to the normal B12 level group. Graphs (b), (c), and (d): Values represent SD change from baseline in log transformed cortisol response per SD increase in maternal exposure variable

In a mixed model analysis, maternal low B12 status, as a binary outcome, was associated with a higher cortisol response to stress (0.36 SD [95% confidence interval (CI): 0.16, 0.57 SD] increase from baseline in peak cortisol response at 30 minutes in maternal low B12 group, compared to the normal B12 group; *P* < .001; Fig. 1; Table 3). Maternal B12 concentration, as a continuous outcome, was negatively associated with cortisol response (–0.13 [–0.22, –0.03] *P* = .01), although the association was nonsignificant when tested at the Bonferroni adjusted significance level of *P* = .001.

**Table 3.** Associations of maternal low B12 status, and maternal B12, folate and homocysteine concentrations with offspring 30-minute cortisol response to the Trier Social Stress Test

Maternal exposure	β (95% CI)	*P*-value
Low B12 status^a^	0.36 (0.16, 0.57)	<.001
B12 concentrations^b^	–0.13 (–0.22, –0.03)	.01
Folate concentrations^b^	0.02 (–0.08, 0.12)	.69
Homocysteine concentrations^b^	0.14 (0.02, 0.26)	.03

*N* = 247. All models describe complete post-Trier Social Stress Test time series (10, 20, 30, 40, and 70 minutes) and are adjusted for offspring age, sex, socioeconomic status, pubertal stage, current BMI, maternal gestational diabetes mellitus status, and maternal BMI during pregnancy.

Abbreviations: BMI, body mass index; CI, confidence interval.

^a^β represents standard deviation (SD) change from baseline in log-transformed cortisol response for maternal low B12 group, compared to normal B12 level

^b^β represents SD change from baseline in log transformed cortisol response per SD increase in maternal exposure variable

Maternal folate concentrations were not associated with cortisol response to stress in the adolescent offspring (Table 3; Fig. 1). There were no significant interactions between maternal B12 and folate concentrations for these associations. Offspring cortisol responses tended to increase with maternal tHcy concentrations (0.14 [95% CI: 0.02, 0.26]; *P* = .03), although again, the association was not significant at the Bonferroni adjusted significance level of *P* = .001 (Table 3; Fig. 1).

### Associations with cardiovascular responses to stress

**Figure 2.**
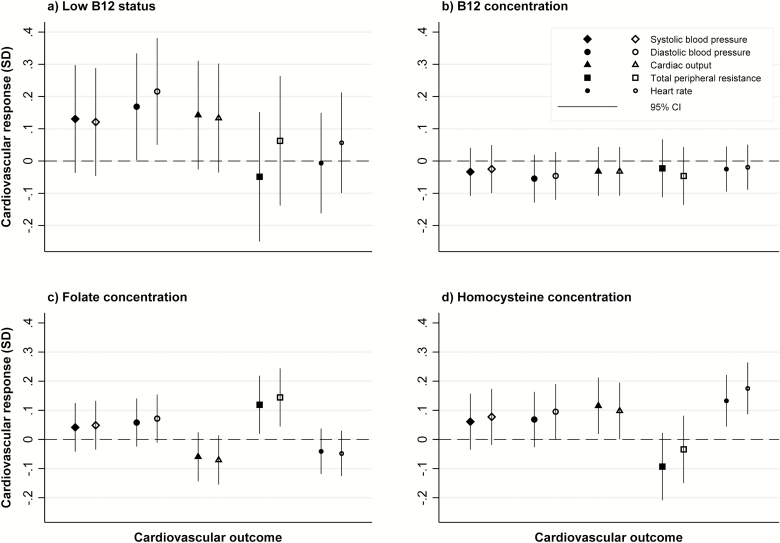
Associations of maternal low B12 status, and maternal B12, folate, and homocysteine concentrations with offspring cardiovascular responses to the Trier Social Stress Test. All graphs: solid markers represent cardiovascular response during the free speech task; open markers represent cardiovascular response during the mental arithmetic task Graph (a): Values represent standard deviation (SD) change from baseline in cardiovascular response for maternal low B12 status group, compared to normal B12 level group. Graphs (b), (c), and (d): Values represent SD change from baseline in cardiovascular response per SD increase in the maternal exposure variable.

Higher maternal tHcy concentration was significantly associated with greater heart rate response to stress during the mental arithmetic task (0.18 [95% CI: 0.09,0.26]; *P* < .001) (Table 4; Fig. 2). Maternal B12 concentration as continuous variable was not associated with offspring cardiovascular stress responses. However, there was a nonsignificant association between maternal B12 categorized as low status and higher diastolic BP during public speaking (*P* = .05) and mental arithmetic tasks (*P* = .01) in the offspring (Table 4; Fig. 2). Offspring born to mothers with higher folate concentrations had a higher TPR response, although the association was nonsignificant after Bonferroni correction (*P* = .02 and .01, respectively, for public speaking and mental arithmetic tasks; Table 4; Fig. 2).

**Table 4.** Associations of maternal low B12 status, and maternal B12, folate, and homocysteine concentrations with offspring cardiovascular responses to the Trier Social Stress Test

Outcome	Low B12 status^a^		B12 concentrations^b^		Folate concentration^b^		Homocysteine concentration^b^	
	β (95% CI)	*P*-value	β (95% CI)	*P*-value	β (95% CI)	*P*-value	β (95% CI)	*P*-value
**At public speaking task**								
Systolic BP	0.13 (–0.04, 0.30)	0.13	–0.03 (–0.11, 0.04)	0.38	0.04 (–0.04, 0.12)	0.33	0.06 (–0.04, 0.16)	0.21
Diastolic BP	0.17 (0.00, 0.33)	0.05	–0.05 (–0.13, 0.02)	0.15	0.06 (–0.02, 0.14)	0.17	0.07 (–0.03, 0.16)	0.16
Heart rate	–0.01 (–0.16, 0.15)	0.94	–0.02 (–0.09, 0.05)	0.49	–0.04 (–0.12, 0.04)	0.31	0.13 (0.04, 0.22)	0.003
Cardiac output	0.14 (–0.03, 0.31)	0.10	–0.03 (–0.11, 0.04)	0.40	–0.06 (–0.14, 0.02)	0.17	0.12 (0.02, 0.21)	0.02
Stroke volume	0.11 (–0.04, 0.27)	0.14	–0.00 (–0.07, 0.06)	0.90	–0.03 (–0.10, 0.05)	0.50	0.04 (–0.05, 0.13)	0.40
Total peripheral resistance	–0.05 (–0.25, 0.15)	0.63	–0.02 (–0.11, 0.07)	0.62	0.12 (0.02, 0.22)	0.02	–0.10 (–0.21, 0.02)	0.11
**At mental arithmetic task**								
Systolic BP	0.12 (–0.05, 0.29)	0.16	–0.03 (–0.10, 0.05)	0.51	0.05 (–0.03, 0.13)	0.25	0.08 (–0.02, 0.17)	0.11
Diastolic BP	0.22 (0.05, 0.38)	0.01	–0.05 (–0.12, 0.03)	0.22	0.07 (–0.01, 0.15)	0.09	0.10 (0.00, 0.19)	0.05
Heart rate	0.06 (–0.10, 0.21)	0.48	–0.02 (–0.09, 0.05)	0.59	–0.05 (–0.13, 0.03)	0.23	0.18 (0.09, 0.26)	<0.001
Cardiac output	0.13 (–0.04, 0.30)	0.12	–0.03 (–0.11, 0.04)	0.41	–0.07 (–0.15, 0.01)	0.10	0.10 (0.00, 0.20)	0.05
Stroke volume	0.07 (–0.08, 0.23)	0.35	–0.01 (–0.08, 0.06)	0.81	–0.03 (–0.11, 0.05)	0.44	–0.01 (–0.10, 0.08)	0.84
Total peripheral resistance	0.06 (–0.14, 0.26)	0.54	–0.05 (–0.14, 0.04)	0.31	0.14 (0.04, 0.24)	0.01	–0.03 (–0.15, 0.08)	0.56

*N* = 229. All models are adjusted for offspring age, sex, socioeconomic status, pubertal stage, current BMI, maternal gestational diabetes mellitus status, and maternal BMI during pregnancy.

Abbreviations: BMI, body mass index; BP, blood pressure; CI, confidence interval.

^a^β represents standard deviation change from baseline in cardiovascular response for maternal low B12 group, compared to normal B12 level.

^b^β represents SD change from baseline in cardiovascular response per SD increase in maternal exposure variable.

All associations were similar in boys and girls, and there were no significant sex interactions for the previously described associations.

## Discussion

To our knowledge, this is the first study of offspring stress responses in relation to measures of maternal nutritional status in humans. In our study, lower B12 status in the pregnant mother was associated with higher cortisol responses to stress in the offspring during adolescence. There was also evidence of an association of higher maternal homocysteine concentrations with greater offspring cardiovascular responses to stress.

Altered neuroendocrine reactivity to stress is thought to increase cardiovascular and mental disorders in humans (1). Impaired nutrition during fetal growth has been suggested to permanently alter physiological stress responses (21). Animal studies support this (22, 23). Langley-Evans et al. showed that protein restriction in rat dams was associated with changes in several indices of HPA axis activity in the fetus (22). There was increased glucocorticoid receptor binding and elevated corticosterone-inducible enzymes in higher brain centers, suggesting increased glucocorticoid sensitivity. A recent study in sheep has demonstrated increased cortisol and adrenal responses in adult offspring of undernourished ewes (23). In humans, studies using birth weight as a proxy for intrauterine nutrition have shown that lower birth weight was associated with higher cortisol (9, 10) and cardiac sympathetic responses to stress in children as well as adults (11, 12). In low- and middle-income countries like India, nutritional deficiencies are common in seemingly healthy pregnant women, and B12 deficiency is particularly common in India, possibly because of vegetarian diets (6). Studies in Pune and Mysore in India, have shown associations of maternal B12, folate, and homocysteine concentrations with neural tube defects (24) and neurocognitive (25, 26) and NCD risk factors in the offspring (6, 14). The current study shows that these nutrients may also predict altered offspring stress mechanisms.

In our study children of mothers with low B12 status had lower baseline cortisol concentrations and a higher stress-induced cortisol increment than offspring of mothers with normal B12 levels. Low B12 status was also associated with greater cardiovascular stress responses, particularly diastolic BP. One-carbon nutrients are vital cofactors in neurodevelopmental processes and deficiencies have been linked to altered neuroendocrine structure and function (4). This includes abnormalities of neural cell proliferation and differentiation, myelination, and synaptogenesis in brain centers associated with higher brain functions (4) that could influence stress perception and reactivity. In addition, low B12 levels may lead to elevated homocysteine, which may damage growing neural cells and affect synaptogenesis by inducing oxidative stress (27). Consistent with this, in our study, higher maternal homocysteine concentrations were also associated with higher cortisol and cardiovascular responses in the adolescent offspring.

Folate also plays an important role in neurodevelopment. Folic acid supplementation is recommended in the preconceptional period to prevent neural tube defects. Yet, in our study, maternal folate concentrations tended to be positively associated with offspring cardiovascular stress responses. We observed nonsignificant positive associations between maternal folate and TPR responses. Increased TPR is one of the mechanisms for elevated BP. In our cohort, mean maternal serum folate concentrations were higher than those reported in other parts of the world (14), and only a few women had low folate status. Higher folate in the presence of B12 deficiency may have adverse neuropsychiatric consequences (28). There is also a suggestion that high folate levels may have adverse implications for health (29). Enzymes that require folate as a cofactor may be inhibited by high levels of folic acid (30). We have shown earlier that higher maternal folate was associated with higher insulin resistance during childhood and adolescence in the Parthenon cohort (14). While optimum micronutrients during the early stages of pregnancy have beneficial effects on fetal development, our findings suggest that exposure to high folate levels in late pregnancy may confer future risk of hypertension in the offspring. This raises an issue for folate supplementation in pregnancy, after the first trimester, especially in the background of high levels of B12 deficiency.

We do not know whether the higher stress responses observed during the TSST-C in our adolescents will increase their future disease risk, but studies in humans support this possibility. A recent meta-analysis showed that greater cardiovascular reactivity, particularly BP responses to laboratory-induced stress, is associated with increased future adverse cardiovascular outcomes (2). Abnormal autonomic cardiac control in children may also have implications for future disease risk (31). The significance of lower baseline cortisol levels in association with low B12 status is less clear. An optimal HPA axis response in anticipation of stress may improve resilience to stressful situations. In preschoolers at risk for antisocial behavior (siblings of juvenile offenders), increased family support resulted in increased pretest salivary cortisol concentrations in relation to a social challenge (32). The authors argued that basal cortisol levels and cortisol responses may have differential associations with risk outcomes. In this context, our results may indicate reduced anticipatory preparation for a stressful challenge in offspring of low B12 mothers, which may have resulted in exaggerated cortisol responses when stressed.

A major strength of our study was the measurement of maternal micronutrients during pregnancy, which is a more robust indicator of maternal nutritional status than dietary or supplement intake levels. This is the first study to use a well-established stress test to examine offspring stress responses in association with maternal nutrient status in humans. A comprehensive range of measurements in the mother and at later follow-up in the offspring enabled relevant adjustments. As B12 concentrations are difficult to interpret in pregnancy due to hemodilution and raised glomerular filtration rate, our values may not be indicative of true levels. We did not measure methylmalonic acid, a specific and sensitive indicator of B12 deficiency, which is a limitation. However, a similar definition of low B12 status has been used in other studies. Other limitations were a lack of data on maternal diet and the use of folic acid and B12 supplements at 30 weeks’ gestation, when maternal nutrient status was measured, and on maternal stress measures.

In conclusion, previous studies from India have shown consistent, although complex, associations between maternal 1-C nutrients and offspring cardiometabolic outcomes during childhood. These findings led to the proposal that nutrient-mediated teratogenesis, in which intrauterine micronutrient deficiencies program permanent structural and functional aberrations, promote increased noncommunicable chronic disease risk in the offspring (7). The novel, although modest, associations observed between 1-C components and offspring stress responses in the current study indicate a plausible mechanism for these prior observations. Replication of these findings in other cohorts is paramount for conclusive evidence, and our group is embarking on testing the role of maternal nutritional status on offspring stress responses in other cohorts in India (33). Although their significance for future disease risk is speculative, our study suggests that children of mothers with low B12 levels are exposed to an increased cardiometabolic risk burden early in the life course. Although long-term follow-up will be required to establish disease risk conclusively, there is some evidence that exaggerated neuroendocrine and cardiovascular responses to stress confer greater risk of cardiovascular disease in later life (2).

## Additional Information

***Disclosure Summary***: Authors have no conflict of interest to disclose

***Data Availability***: Restrictions apply to the availability of data generated or analyzed during this study to preserve patient confidentiality or because they were used under license. The corresponding author will on request detail the restrictions and any conditions under which access to some data may be provided.
